# Safety and preliminary immunogenicity of JNJ-64041809, a live-attenuated, double-deleted *Listeria monocytogenes*-based immunotherapy, in metastatic castration-resistant prostate cancer

**DOI:** 10.1038/s41391-021-00402-8

**Published:** 2021-07-13

**Authors:** Charles G. Drake, Russell K. Pachynski, Sumit K. Subudhi, Douglas G. McNeel, Emmanuel S. Antonarakis, Todd M. Bauer, Peter Lauer, Dirk Brockstedt, Daniel Patricia, Mark Wade, Enrique Zudaire, Nibedita Bandyopadhyay, Dolly A. Parasrampuria, Suzette Girgis, Gary E. Mason, Roland E. Knoblauch, Nicole Stone, Jeffrey R. Infante, Marco M. Gottardis, Lawrence Fong

**Affiliations:** 1 Columbia University Medical Center, New York, NY USA; 2 Washington University of St. Louis, St. Louis, MO USA; 3 The University of Texas MD Anderson Cancer Center, Houston, TX USA; 4 University of Wisconsin Carbone Cancer Center, Madison, WI USA; 5 Johns Hopkins Kimmel Medical Institute, Baltimore, MD USA; 6 Sarah Cannon Research Institute/Tennessee Oncology, PLLC, Nashville, TN USA; 7 Aduro Biotech, Berkeley, CA USA; 8 Janssen Research & Development, Spring House, PA USA; 9 Janssen Research & Development, Raritan, NJ USA; 10 UCSF Helen Diller Family Comprehensive Cancer Center, San Francisco, CA USA; 11 Present Address: Janssen Research & Development, Spring House, PA USA

**Keywords:** Prostate cancer, Cancer therapy

## Abstract

**Background:**

The safety and immunogenicity of JNJ-64041809 (JNJ-809), a live-attenuated, double-deleted *Listeria monocytogenes* (LADD *Lm*)-based immunotherapy targeting 4 relevant prostate cancer antigens, was evaluated in a phase 1 study in patients with metastatic castration-resistant prostate cancer (mCRPC).

**Methods:**

Men with progressive mCRPC who had received ≥2 prior approved therapies were enrolled. Primary study objectives were to determine the recommended phase 2 dose (RP2D) and to evaluate the safety and immunogenicity of JNJ-809.

**Results:**

A total of 26 patients received JNJ-809 (1 × 10^8^ CFU (*n* = 6); 1 × 10^9^ CFU (*n* = 20)). No dose-limiting toxicities were reported, and 1 × 10^9^ CFU was selected as the RP2D. The most common adverse events (AEs) reported were chills (92%), pyrexia (81%), and fatigue (62%). The most frequent grade ≥3 AEs were lymphopenia (27%) and hypertension (23%). Serious AEs were reported in 27% of patients including 1 patient with grade 3 intestinal obstruction. JNJ-809 transiently induced peripheral cytokines, including interferon-γ, interleukin-10, and tumor necrosis factor-α. Of the 7 patients evaluable for T cell responses at the 1 × 10^9^ CFU dose, evidence of post-treatment antigenic responses were observed in 6 to the *Listeria* antigen listeriolysin O and in 5 to ≥1 of the 4 encoded tumor antigens. Best overall response was stable disease in 13/25 response-evaluable patients. The study was terminated early as data collected were considered sufficient to evaluate safety and immunogenicity.

**Conclusions:**

JNJ-809 has manageable safety consistent with other LADD *Lm*-based therapies. Limited antigen-specific immune responses were observed, which did not translate into objective clinical responses.

## Introduction

Prostate cancer is among the leading causes of new cancer diagnoses (7.1%) and the fifth leading cause of cancer death in men (3.8%) worldwide [1]. Localized prostate cancer is managed by active surveillance for patients with low-risk disease, higher risk localized disease is treated with either radical prostatectomy or radiation therapy. These treatments can be curative for some patients, but approximately 20–40% of men who undergo radical prostatectomy and 30–50% of men treated with radiation therapy will develop biochemical recurrence [2, 3]. Salvage radiotherapy/prostatectomy and/or androgen deprivation therapy are viable treatment options for biochemically recurrent prostate cancer [4]; however, the majority of patients will eventually develop metastatic castration-resistant prostate cancer (mCRPC). Patients can also develop de novo metastatic castration-sensitive prostate cancer, a more aggressive cancer that is associated with worse prognosis as compared with prostate cancer that metastasizes after initial diagnosis [5, 6]. Next-generation hormonal therapies (NHTs) have improved clinical outcomes in mCRPC [7–10], but as with other treatments, NHTs are limited by primary or acquired resistance [11–13], highlighting the need for novel effective therapies.

Immunotherapeutic approaches to treating prostate cancer have had limited success. Currently, two immunotherapies are approved for the treatment of prostate cancer: sipuleucel-T, an autologous cellular immunotherapy targeting prostatic acid phosphatase (PAP) for the treatment of asymptomatic or minimally symptomatic mCRPC and pembrolizumab, an immune checkpoint inhibitor (ICI) for treatment of microsatellite instability-high or mismatch repair-deficient prostate cancer in patients who have exhausted available therapies [14, 15]. A challenge in developing immunotherapies for prostate cancer has been the multiple immune evasion mechanisms, including decreased major histocompatibility complex (MHC) class I expression and increased infiltration of regulatory T cells (Tregs) and myeloid-derived suppressor cells (MDSCs) [16–19]. Given the immunosuppressive tumor microenvironment of “cold” tumors such as prostate cancer tumors, response to ICIs has been poor. In two phase 3 studies of ipilimumab in patients with prostate cancer, no survival benefit over placebo was observed [20, 21], and in a phase 1 study of nivolumab in CRPC, no objective responses were reported [22].

The live-attenuated double-deleted (LADD) *Listeria monocytogenes* (*Lm*) platform can encode and deliver multiple heterologous tumor antigens to induce innate and adaptive immune responses [23]. The deletion of the genes encoding the actin assembly-inducing protein (actA) and internalin B from the *Lm* chromosome leads to 1/1000 of the virulence without decreasing antigenicity [23]. *Lm*-encoded antigens are processed via major MHC class I and class II antigen-processing pathways. Additionally, *Lm*-based immunotherapies can inhibit the immunosuppressive efficacy of Tregs and MDSCs in the tumor microenvironment [24–26]. In preclinical models, *Lm*-based vaccination drives antigen-specific T cell expansion and remodeling of the tumor microenvironment [27] without upregulation of the inhibitory PD-1 checkpoint molecule (Nirschl and Drake, to be submitted for publication). Taken together, LADD *Lm*-based therapeutics have the potential to deliver tumor-specific antigens, enhance immune responses, and overcome immune evasion mechanisms in mCRPC and other tumors [26].

JNJ-64041809 (JNJ-809) is a LADD *Lm*-based immunotherapy that encodes and expresses 4 antigens relevant to prostate cancer: prostatic acid phosphatase (PAP) [28], prostate-specific membrane antigen (PSMA) [29], synovial sarcoma X breakpoint 2 (SSX2) [30, 31], and homeobox protein NKX3.1 [32–34]. With regards to these antigens, PAP is secreted by both benign and malignant prostate columnar epithelium cells and is the target antigen for sipuleucel-T [35]. PSMA is highly expressed in prostate cancers, and the increased expression of PSMA correlates with higher grade malignancies, metastatic disease, and CRPC [29]. SSX2, a prostate cancer-testis antigen, is relatively overexpressed in metastatic prostate cancer as compared with localized prostate cancer or healthy prostate samples [30, 31]. The potential of SSX2 as an immune target is supported by immunoglobulin G (IgG) responses and CD8+ T cells specific for SSX2 observed in patients with prostate cancer [30, 31]. The homeobox protein NKX3.1 is involved in the development, differentiation, and function of the prostate [32–34]. NKX3.1 is a marker of castration-resistant luminal epithelial cells and is required for the emergence of CRPC in animal models [34]. These data suggest that castration-resistant NKX3.1-expressing cells may serve as stem cells and give rise to advanced disease [34, 36]. To evaluate the safety and immunogenicity of JNJ-809, we conducted a first-in-human phase 1 study in patients with mCRPC.

## Methods

### Study design

The study protocol and amendments were approved by the institutional review board at each of the sites. The study was conducted in accordance with the ethical principles that have their origin in the Declaration of Helsinki and that are consistent with Good Clinical Practices and applicable regulatory requirements. All patients or their legally acceptable representatives provided written consent to participate in the study after having been informed about the nature and purpose of the study, participation/termination conditions, and risks and benefits of treatment. This study was registered at ClinicalTrials.gov, NCT02625857.

This was a first-in-human, phase 1, open-label, multicenter, two-part study in patients with mCRPC. Key eligibility criteria included men aged 18 years or older who had histologically confirmed metastatic prostate cancer; received at least 2 prior therapies in the castration-resistant setting; ongoing androgen deprivation therapy; serum testosterone levels <50 ng/dL within 4 weeks prior to start of study drug; Eastern Cooperative Oncology Group (ECOG) performance status of 0–1; adequate baseline organ function; and no history of major implants or devices (added in a protocol amendment). For cohort 2B, patients were required to have a primary tumor or metastatic lesion(s) amenable to tumor biopsies.

The primary objective of part 1 dose escalation was to determine the recommended phase 2 dose (RP2D) of JNJ-809. The primary objective of part 2 dose expansion was to characterize the safety and immunological efficacy of JNJ-809 at the RP2D in 2 expansion cohorts: cohort 2A (mCRPC) and cohort 2B (mCRPC with lesions amenable to tumor biopsies). Key secondary objectives included evaluating preliminary clinical efficacy and assessing the blood culture and shedding profile of JNJ-809; exploratory objectives included evaluating other aspects of immunologic efficacy and pharmacodynamic biomarkers.

Two dose levels of JNJ-809, intravenous 1 × 10^8^ or 1 × 10^9^ colony-forming units (CFUs) once every 21 days, were explored sequentially in part 1 using a 3 + 3 design. The doses were selected based on previous clinical experience with CRS-207, another *Lm*-based vaccine expressing human mesothelin, where the 1 × 10^8^ and 1 × 10^9^ CFU doses were tolerable, and 1 × 10^9^ CFU was identified as the maximum tolerated dose after a dose-limiting toxicity (DLT) was reported at the 1 × 10^10^ CFU dose [37]. Selection of the RP2D was based on safety and pharmacodynamic assessments of innate immune responses, including lymphocyte counts and cytokine release. In part 2 dose expansion, cohorts 2A and 2B were administered JNJ-809 at the RP2D. Patients continued to receive treatment until (1) both prostate-specific antigen progression and radiographic progression were documented, or (2) clinical progression, or (3) physician’s decision to start new anti-cancer therapy. Progression was based on Prostate Cancer Clinical Trials Working Group 2 (PCWG2) criteria [38]

### Study evaluations

Safety was assessed by physical examinations, ECOG performance status, laboratory tests, vital signs, electrocardiograms, AEs, and concomitant medication usage. The DLT evaluation period was the first 21 days after the start of the first infusion. AEs were graded according to the National Cancer Institute Common Terminology Criteria for Adverse Events, v. 4.03.

Efficacy was assessed by the investigator according to (PCWG2) criteria [38] and Response Evaluation Criteria in Solid Tumors (RECIST), v. 1.1.

Peripheral blood samples were collected to evaluate the pharmacokinetics of JNJ-809 at cycle 1 day 1 (prior to infusion), 2, 4, 24, and 48 h after the end of the first infusion, cycle 1 day 7, cycle 1 day 14, cycle 2 day 1 (prior to infusion), and prior to each infusion in which a disease assessment occurred. Feces, urine, and saliva were collected at similar time points to the pharmacokinetic samples above and at the end of treatment and follow-up visits to assess potential shedding of JNJ-809. Patients were required to receive prophylactic antibiotics (intravenous amoxicillin 500 mg thrice daily (or oral trimethoprim 160 mg and sulfamethoxazole 800 mg twice daily for patients with penicillin allergy)) for 7 days after discontinuation of treatment.

Biomarker analyses, including interferon-γ (IFN-γ) enzyme-linked immunospot (ELISpot) and flow cytometry for intracellular cytokine staining and markers of T cell activation, were performed using blood samples. Peripheral blood mononuclear cell (PBMC) collection and isolation were performed at each clinical site while antigen-specific T cell responses were evaluated centrally. The predefined quality control for ELISpot analysis was performed centrally and based on sufficient number of cells for the analysis, available baseline and at least one post-treatment sample from each patient, and a minimum viability threshold of 75% and recovery threshold of 50%. Samples that did not meet these criteria were excluded from the analysis. The positivity cutoff for ELISpot analysis was defined as the background subtracted response at any point following immunotherapy minus the background subtracted response at screening being greater than 1.5 times the standard deviation of the baseline antigen-specific response or at least 10 spot-forming units per 10^6^ PBMC or greater. To evaluate induction of an antitumor response, pre- and post-treatment tumor biopsies of metastatic lesions were stained by immunohistochemistry (IHC) for the expression of markers associated with immune infiltrate using relevant assays.

### Statistical methods

The all-treated analysis population consisted of those patients who received at least 1 dose of study agent; this population was used for all efficacy and safety analyses. The biomarker analysis population consisted of all patients who received at least 1 dose of study agent and had at least 1 pre- and post-treatment evaluable biomarker measurement.

For part 1, up to 10 patients were to be treated at each dose. For part 2, approximately 20 and 10 patients were to be enrolled in cohorts 2A and 2B, respectively. A sample size of 20 would provide a two-sided 95% confidence interval of 27–73% assuming that at least 50% of patients developed a relevant antigen-specific T cell response.

Data were summarized using descriptive statistics. Continuous variables included number of observations, mean, standard deviation, median, and range. Categorical values were summarized using number of observations and percentages.

## Results

Between 16 December 2015 and 3 July 2018, 26 men with mCRPC were enrolled in the study; 12 patients in part 1 (6 at 1 × 10^8^ CFU and 6 at 1 × 10^9^ CFU) and 14 patients in part 2 (6 in cohort 2A and 8 in cohort 2B). The median age was 67 years (range, 46–85), and the majority of patients were white (96%; Table 1). The median time from initial diagnosis to first dose of study agent was 6 years (range, 1–18). The majority of patients had Gleason score ≥8 (62%), 27% had bone-only disease, and 62% had soft tissue or node disease (Table 1). Seventeen patients had received prior first-generation androgen receptor therapies (bicalutamide, nilutamide, flutamide), 3 patients had received prior second-generation or later androgen receptor therapy (abiraterone or enzalutamide), and 4 had received prior taxane therapy.

**Table 1:** Demographic and baseline disease characteristics.

	1 × 10^8^ CFU *n* = 6	1 × 10^9^ CFU *n* = 20	Total *n* = 26
Median age, years (range)	74 (58–77)	65 (46–85)	67 (46–85)
Race, *n* (%)			
White	5 (83)	20 (100)	25 (96)
Black or African American	1 (17)	0	1 (4)
ECOG PS, *n* (%)			
0	2 (33)	7 (35)	9 (35)
1	4 (67)	13 (65)	17 (65)
Time from initial diagnosis, years (range)	5 (1–18)	6 (1–17)	6 (1–18)
Gleason score, *n* (%)			
<7	1 (17)	2 (10)	3 (12)
7	2 (33)	3 (15)	5 (19)
≥8	3 (50)	13 (65)	16 (62)
Unknown	0	2 (10)	2 (8)
Extent of disease at entry, *n* (%)			
Bone	4 (67)	19 (95)	23 (89)
Bone only	1 (17)	6 (30)	7 (27)
Soft tissue or node	2 (33)	14 (70)	16 (62)
Other	3 (50)	5 (25)	8 (31)
Evidence of disease progression, *n* (%)			
PSA	0	14 (74)	14 (56)
Radiographic	6 (100)	11 (58)	17 (68)
Laboratory parameters			
Median hemoglobin, g/dL (range)	10 (9–13)	11 (9–14)	11 (9–14)
Median alkaline phosphatase, U/L (range)	178 (74–633)	88 (25–1015)	96 (25–1015)
Median lactate dehydrogenase, U/L (range)	849 (314–2719)	370 (99–2203)	401 (99–2719)

*CFU* colony-forming unit, *ECOG PS* Eastern Cooperative Oncology Group performance status, *PSA* prostate-specific antigen.

All 26 patients discontinued study treatment. Progressive disease was the most common reason for discontinuation (81%), followed by patient withdrawal (12%), AE (4%), and treatment discontinuation because of major indwelling hardware implant, which was a new exclusion criterion in the amended protocol (4%; Supplementary Fig. 1). The median duration of study treatment was 2.5 months (range, 0–10). The study was terminated early as data collected from 26 patients was sufficient to evaluate safety and immunogenicity of JNJ-809.

No DLTs were reported during dose escalation, and 1 × 10^9^ CFU was selected as the RP2D. AEs were consistent across both parts 1 and 2, and all patients reported AEs that were related to study treatment (Table 2). One patient experienced grade 3 small intestinal obstruction which led to discontinuation of study treatment; the small intestinal obstruction was not related to study treatment. The most frequently reported AEs were chills (92%), pyrexia (81%), and fatigue (62%; Table 2). The chills and pyrexia, which resolved within 48 h and were consistent with transient activation of the innate immune response to JNJ-809, were managed with supportive care during the required observation period in the outpatient setting. Grade ≥3 AEs were reported in 69% of patients; lymphopenia (27%) and hypertension (23%) were most frequent and were generally transient, resolving within 1–4 days. Seven (27%) patients had serious AEs, including 1 patient with pelvic pain, penile pain, and rectal pain and another patient with large intestinal obstruction and large intestine perforation, with the latter being assessed by the investigator as related to JNJ-809. However, the sponsor considered these events not related to study treatment because of the patient’s history of prior radiation to the area, and the large intestine perforation occurred in the setting of colonic stent erosion with diffuse peritonitis. The other 5 patients had a single serious AE of spinal cord compression, gastrointestinal hemorrhage, confusional state, small intestinal obstruction, or neuralgia.

**Table 2:** Adverse events.

AEs, *n* (%)	1 × 10^8^ CFU *n* = 6	1 × 10^9^ CFU *n* = 20	Total *n* = 26
Any AEs	6 (100)	20 (100)	26 (100)
Related AEs	6 (100)	20 (100)	26 (100)
Grade ≥3 AEs	5 (83)	13 (65)	18 (69)
Related grade ≥3 AEs	3 (50)	8 (40)	11 (42)
Serious AEs	2 (33)	5 (25)	7 (27)
Related serious AEs	0	1 (5)	1 (4)
Grade ≥3 serious AEs	2 (33)	4 (20)	6 (23)
AEs leading to treatment discontinuation	0	1 (5)	1 (4)
Most common AEs (≥20% of total), *n* (%)			
Chills	5 (83)	19 (95)	24 (92)
Pyrexia	5 (83)	16 (80)	21 (81)
Fatigue	4 (67)	12 (60)	16 (62)
Nausea	3 (50)	9 (45)	12 (46)
Vomiting	2 (33)	9 (45)	11 (42)
Anemia	4 (67)	7 (35)	11 (42)
Arthralgia	3 (50)	7 (35)	10 (39)
Decreased appetite	1 (17)	7 (35)	8 (31)
Constipation	3 (50)	4 (20)	7 (27)
Back pain	2 (33)	5 (25)	7 (27)
Lymphopenia	1 (17)	6 (30)	7 (27)
Hypertension	1 (17)	6 (30)	7 (27)
Diarrhea	3 (50)	3 (15)	6 (23)
Headache	1 (17)	5 (25)	6 (23)

*AE* adverse event, *CFU* colony-forming unit.

LADD *Lm* bacteremia was monitored by both aerobic and anaerobic cultures of blood samples at specified time points following infusion. All 6 patients who received 1 × 10^8^ CFU had negative blood cultures for LADD *Lm* at each time point tested. For the 20 patients treated with 1 × 10^9^ CFU, at cycle 1 day 1, 2 h postdose, 4 patients had both aerobic and anaerobic blood cultures that were positive for LADD *Lm* 2 h postdose, and 1 patient had only a positive aerobic blood culture and another patient had only a positive anaerobic blood culture. At 4 h postdose, only 1 patient had an aerobic blood culture that remained positive for LADD *Lm*. However, all subsequent blood cultures were negative for LADD *Lm* for all patients. No fecal, urine, or saliva samples were positive for LADD *Lm*.

Twenty-five of the 26 enrolled patients had measurable disease at baseline and could be assessed for objective response by RECIST. The best overall response was stable disease (SD) in 13 of 25 (52%) response-evaluable patients; 11 patients had SD for ≥12 weeks, and 6 patients had SD for ≥16 weeks (Supplementary Fig. S2). Nine (36%) patients had progressive disease, and 3 (12%) were not evaluable due to the lack of post-treatment response assessment (Table 3).

**Table 3:** Best overall response.

Best overall response, *n* (%)	1 × 10^8^ CFU *n* = 6	1 × 10^9^ CFU *n* = 19	Total *n* = 25^a^
Stable disease	3 (50)	10 (53)	13 (52)
Progressive disease	2 (33)	7 (37)	9 (36)
Non-evaluable^b^	1 (17)	2 (11)	3 (12)

^a^Only 25 patients had measurable disease at baseline and were evaluable for response per RECIST.

^b^Three patients were not evaluable by RECIST due to the absence of radiographic progression.

*CFU* colony-forming unit.

Of the 7 paired biopsies that were collected, 3 did not have sufficient tumor tissue for analysis (in the pre- and/or post-treatment biopsies), and 4 were evaluable for CD8 and FOXP3 IHC staining. In 3 pairs of pre- and post-treatment biopsies, CD8+ cells and FOXP3+ cells either decreased, remained unchanged, or increased. In one biopsy pair, CD8+ cells increased and FOXP3+ cells were unchanged. Results were considered inconclusive, given the variability and the low number of evaluable samples available for analysis.

**Fig. 1:**
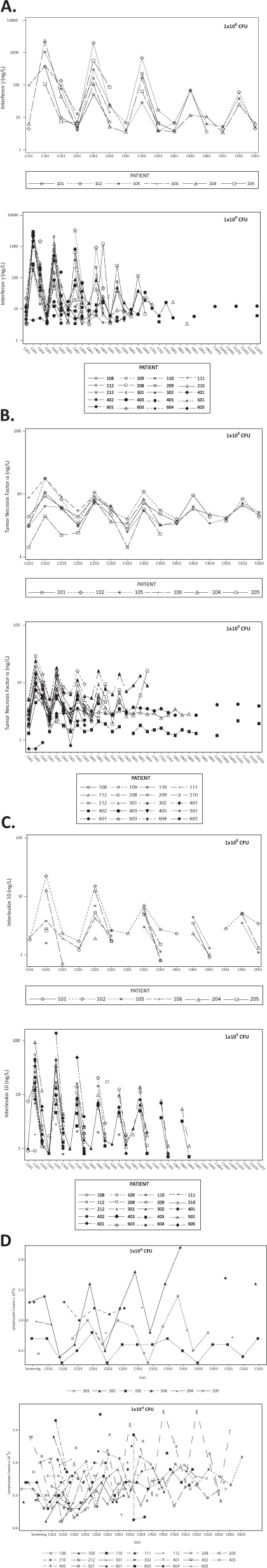
Release of serum cytokines following JNJ-809 infusion. Transient increases in pro-inflammatory serum cytokines: **A** IFN-γ, **B** TNFα, and **C** IL-10 were observed after treatment with both 1 × 10^8^ CFU (top) and 1 × 10^9^ CFU (bottom) doses of JNJ-809. **D** Lymphocyte activation was observed following treatment with JNJ-809 at 1 × 10^8^ CFU (top) and 1 × 10^9^ CFU (bottom) doses. CFU = colony-forming unit; IFN = interferon; IL-10 = interleukin-10; TNF = tumor necrosis factor.

Innate immune responses were demonstrated in all patients at both doses. Transient increases in levels of serum pro-inflammatory cytokines IFN-γ, tumor necrosis factor alpha (TNFα), and interleukin-10 (IL-10) were greatest 24 h after JNJ-809 infusion and returned to baseline by 48 h after infusion (Fig. 1A–C). Lymphocyte activation was also observed after treatment with JNJ-809 (Fig. 1D).

**Fig. 2:**
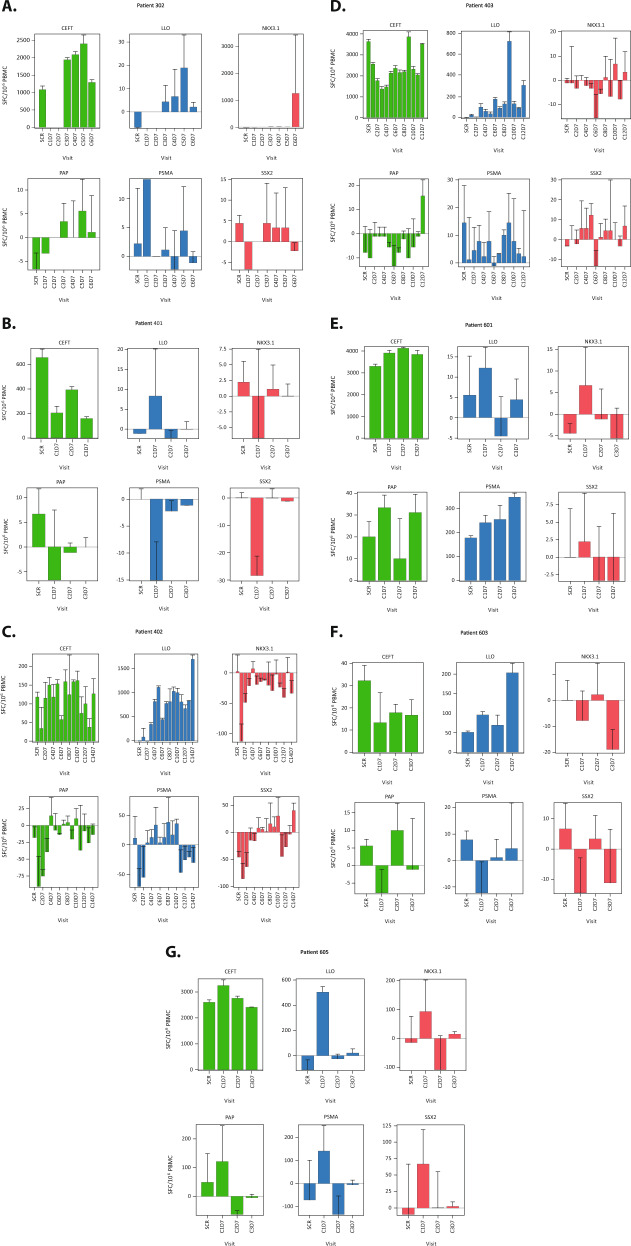
ELISpot analysis of tumor-associated antigens. ELISpot analyses are shown for all 7 patients (**A**–**G**) who had evaluable samples. Error bars represent the standard deviation of 3 replicates for each ELISpot. C = cycle; CEFT = cytomegalovirus, Epstein-Barr virus, influenza, and tetanus toxoid; D = day; LLO = listeriolysin O; PAP = prostatic acid phosphatase; PBMC = peripheral blood mononuclear cell; PSMA = prostate-specific membrane antigen; SCR = screening; SFC = spot forming colonies; SSX-2 = synovial sarcoma X chromosome breakpoint-2.

Adaptive immune responses were assessed by quantifying antigen-specific T cell responses using ELISpot analysis. Seven of the 26 patients had PBMCs that met the predefined quality-control criteria for ELISpot analysis. All 7 patients showed T cell responses to the cytomegalovirus, Epstein-Barr virus, influenza, and tetanus toxoid (CEFT) positive control epitopes, and 6 patients demonstrated reactivity to the *Listeria* antigen listeriolysin O (LLO) in at least one post-treatment assessment (Fig. 2). Post-treatment T cell responses to at least 1 of the 4 encoded tumor-associated antigens were observed in 5 patients, with no specific tumor antigen demonstrating superior responses over the others (Fig. 2). Although the antigen-specific T cell responses were variable in magnitude and persistence, they were consistently lower than the responses to LLO and CEFT (Fig. 2).

## Discussion

In this first-in-human study of JNJ-809 in men with mCRPC, the safety profiles of the 1 × 10^8^ and 1 × 10^9^ CFU doses were comparable and consistent with those reported for LADD-based immunotherapeutics [37, 39, 40]. Across both doses, SD was the best overall response achieved among the response-evaluable patients.

The most commonly reported AEs were chills (92%) and pyrexia (81%), the severity and incidence of which did not appear to be associated with JNJ-809 dose, although this comparison is limited by the small number of patients in the study. One patient had serious adverse events of large intestinal obstruction and large intestine perforation which were considered to be study drug-related by the investigator. However, because the large intestine perforation was observed in the context of erosion of stents placed to treat colonic obstruction, the sponsor considered these events not related to study treatment.

The risk of LADD *Lm* bacteremia was evaluated through blood cultures and shedding samples. All 6 patients who received JNJ-809 doses of 1 × 10^8^ CFU had negative blood cultures for LADD *Lm* at each time point tested. A number of patients who received JNJ-809 doses of 1 × 10^9^ CFU had transient positive blood cultures on the day of drug administration, but all subsequent blood cultures from these patients were negative for LADD *Lm*. Additionally, all blood cultures tested at the end of treatment (before the start of the required post-treatment antibiotic therapy) and at long-term follow-up (every 3 months after the end of treatment) were negative for LADD *Lm*. None of the fecal, urine, or saliva samples from patients treated at either dose tested positive for LADD *Lm*. These data suggest that although JNJ-809 is administered intravenously, the bacteria are cleared from the systemic circulation quickly, and the risk of persistent bacteremia or inter-person transmission is low. The risk is further lowered with a required course of antibiotics and for those patients with a central venous catheter (e.g., Port-a-Cath or Mediport), the first dose of antibiotics was given through the port.

Consistent with reports of other LADD therapeutics [40], cytokine and chemokine release after JNJ-809 administration were transient and peaked approximately 24 h after the end of infusion before returning to baseline levels within 48–72 h. This cytokine release profile coincided with reported chills and pyrexia, which resolved within 48 h and was consistent with activation of innate immunity.

ELISpot biomarker data were limited (7 of 26 patients) due to inadequate quality of PBMC processing at some sites. Specifically, a number of samples failed to meet the predefined quality-control criteria based on cell viability and recovery, and issues related to sample handling and processing were identified centrally when samples were processed for ELISpot analysis. This highlights the importance of ensuring sites are proficient in PBMC processing and establishing pre-analytical sample quality controls to allow for timely identification of potential sample quality issues at the site level and implementation of corrective measures. Although the number of samples was small and ELISpot responses were variable in magnitude and persistence, the results indicate that antigen-specific T cell responses to the JNJ-809-encoded antigen LLO can be reliably elicited.

The study was terminated early as data collected from the 26 patients enrolled in the study were sufficient to evaluate safety and immunogenicity. During the course of the study, a Janssen clinical hold was placed on the LADD platform due to concerns of *Listeria* persistence in another Janssen-sponsored LADD study [40]. Additionally, in this study, ELISpot analysis failed to demonstrate robust evidence of adaptive immune response either due to improperly processed samples or failure of JNJ-809 to elicit a response to targeted prostate cancer antigens, despite the ability to elicit LLO responses. Based on the lack of immunogenic and clinical response data up until that point, and in the context of other Janssen-sponsored LADD studies, the strategic decision was made to stop the study due to the low likelihood of demonstrating the primary endpoint.

In conclusion, the safety profile of JNJ-809 was similar across both doses evaluated and is consistent with other LADD-based therapeutics [37, 39]. Activation of innate and acquired immune responses were observed following JNJ-809 monotherapy; however, the sample size was relatively small, and the magnitude of immune responses was modest with no clear clinical benefit demonstrated for monotherapy. Having demonstrated safety, intervention at earlier stages of cancer may result in more robust responses.

## Supplementary information

Supplemental material.
