# Supplementary material for: Safety and preliminary immunogenicity of JNJ-64041809, a live-attenuated, double-deleted Listeria monocytogenes-based immunotherapy, in metastatic castration-resistant prostate cancer
Source: Prostate Cancer Prostatic Dis. 2021 Jul 13;25(2):219–28. doi: 10.1038/s41391-021-00402-8 (PMC9184270; doi:10.1038/s41391-021-00402-8)

**Supplementary Figure S1. Patient Flow Chart**

**Enrolled**

**N=26**

**Part 1: Dose Escalation**

**n=12**

**Part 2: Dose Expansion**

**n=14**

**Analyzed**

**N=26**

**Discontinued Treatment**

**N=26**

Progressive disease (n=21)

Withdrawal by patient (n=3)

Adverse event (n=1)

Other^a^ (n=1)

**JNJ-809 1x10^8^ CFU**

**n=6**

**JNJ-809 1x10^9^ CFU**

**n=6**

**JNJ-809 1x10^9^ CFU**

**n=14**

^a^Patient stopped treatment after adoption of a protocol amendment that prevented the patient from meeting the new exclusion criteria (possession of a major indwelling implant).

**Supplementary Figure S2. Radiographic Progression-free Survival**


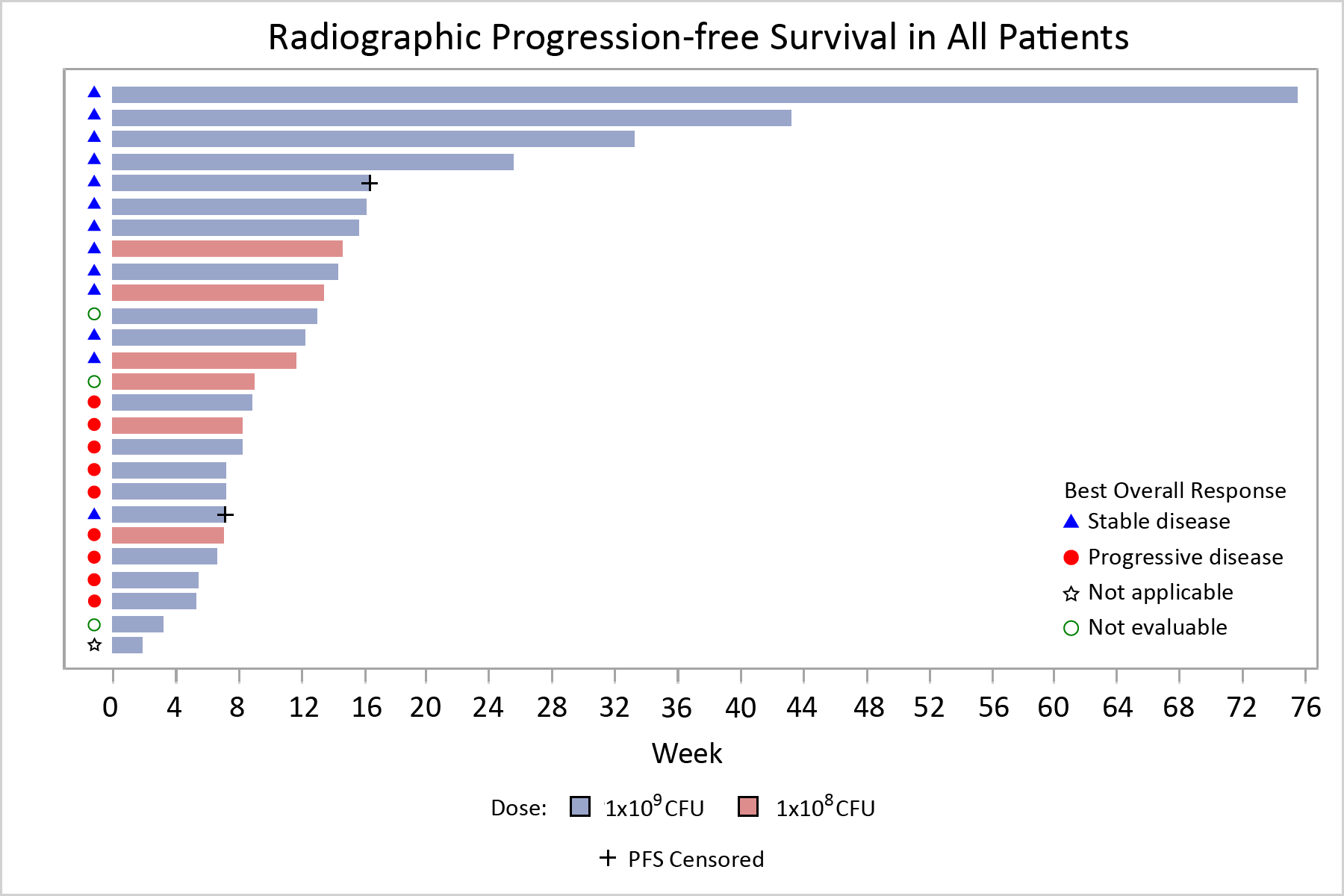

Supplement: Supplementary file 1 — Supplemental material. [file 41391_2021_402_MOESM1_ESM.docx]
